# Experiencia en educación sanitaria por Whatsapp en una población desfavorecida de Pampas - Perú

**DOI:** 10.1016/j.aprim.2022.102347

**Published:** 2022-05-04

**Authors:** Liszeth Paola Cerna Ruiz, Mercedes Acosta Román, Marisel Roxana Valenzuela Ramos

**Affiliations:** a Universidad Nacional de Tayacaja, Pampas, Perú; b Universidad Nacional de Piura, Perú

Con el inicio de la pandemia COVID-19 muchas instituciones se vieron afectadas, incluidas aquellas que brindaban servicios a la comunidad como la promoción de la salud, las intervenciones educativas, los programas educativos y las enseñanzas de determinados temas enfocados en los cuidados de la salud. Las enseñanzas dirigidas a las embarazadas, por lo general, se basan en el apoyo prenatal, educación y consejería. Las evidencias demuestran que aquellas embarazadas que reciben educación durante la etapa prenatal dan a luz con mayor facilidad y asumen con mejor la responsabilidad del cuidado de sus recién nacidos^1^. Encontrándose más preparadas para iniciar una nueva etapa en la vida, en conjunto con el nuevo ser, ya que la educación prepara a los padres para el embarazo, el parto y el periodo posnatal; se aconseja que esto se lleve a cabo durante las semanas 20 y 30^2^. En los cuidados del recién nacido se debe incorporar la educación acerca de la higiene bucal, la cual debe motivar y sensibilizar a la madre. Los programas, las intervenciones educativas se suelen realizar durante la presencialidad y, sobre todo, en las zonas más desfavorecidas. Generalmente ante este hecho se hace presente el personal de salud al cual pertenece la población al ser intervenida. Pero en el contexto de COVID-19 esta labor realizada por los profesionales de salud se vio suspendida. Ante esta nueva realidad, y para dar cumplimiento a las nuevas normas establecidas por el estado peruano, en Huancavelica, y en especial en el Distrito de Pampas, más de la mitad de la población vive en pobreza o extrema pobreza^3^, a pesar de que existen múltiples programas de ayuda a la población. Los niños, ancianos y mujeres embarazadas son considerados como una población vulnerable y son el centro de atención para poner en marcha la motivación, concientización y sensibilización de los programas educativos. Por lo expuesto, el objetivo de la investigación fue conocer la experiencia de una de una investigación-acción participativa sobre educación en salud dirigido a embarazadas y madres de niños en edad preescolar en el contexto COVID-19. Se trata de una investigación cualitativa de diseño acción participativa. El estudio se realizó en el Distrito de Pampas, perteneciente a la provincia de Tayacaja, ubicado en la región de Huancavelica.

Participantes: el estudio estuvo conformado por 20 participantes entre embarazadas y madres de niños en edad preescolar. Fueron realizadas cuatro sesiones educativas, la primera sesión trató de la alimentación en niños en edad preescolar, la mochila de emergencia, los recursos naturales y la higiene oral. Las cuatro sesiones se desarrollaron de manera virtual a través de la aplicación WhatsApp, para el reforzamiento de las sesiones se llevó a cabo una sesión presencial con el fin de reforzar los conocimientos adquiridos y motivar a las participantes.

Los resultados obtenidos fueron satisfactorios, las participantes mostraron su agradecimiento, además de manifestar que las actividades desarrolladas serían de provecho para ellas y sus familias. En las sesiones educativas se logra que la enseñanza-aprendizaje se efectúe de manera óptima, ya que tanto el operador como el participante aprenden uno del otro dialogando y practicando. Tanto para los preescolares, como para embarazadas y madres que dan de lactar, utilizando alimentos variados en el Distrito de Pampas.

## Conflicto de intereses

Los autores declaramos no tener conflicto de intereses
